# Characterization of a Recombinant Thermostable Newcastle Disease Virus (NDV) Expressing Glycoprotein gB of Infectious Laryngotracheitis Virus (ILTV) Protects Chickens against ILTV Challenge

**DOI:** 10.3390/v15020500

**Published:** 2023-02-11

**Authors:** Zhe Zeng, Yan He, Zichen Wang, Lun Yao, Li Li, Yu Shang, Hongcai Wang, Rongrong Zhang, Huabin Shao, Qingping Luo, Guoyuan Wen

**Affiliations:** 1 Key Laboratory of Prevention and Control Agents for Animal Bacteriosis (Ministry of Agriculture and Rural Affairs), Institute of Animal Husbandry and Veterinary, Hubei Academy of Agricultural Sciences, Wuhan 430064, China; 2 Hubei Provincial Key Laboratory of Animal Pathogenic Microbiology, Institute of Animal Husbandry and Veterinary, Hubei Academy of Agricultural Sciences, Wuhan 430064, China; 3 Hubei Hongshan Laboratory, Wuhan 430064, China

**Keywords:** Newcastle disease virus, glycoprotein gB, infectious laryngotracheitis virus, vector vaccine, thermostability

## Abstract

Infectious laryngotracheitis (ILT) and Newcastle disease (ND) are two important avian diseases that have caused huge economic losses to the poultry industry worldwide. Newcastle disease virus (NDV) has been used as a vector in the development of vaccines and gene delivery. In the present study, we generated a thermostable recombinant NDV (rNDV) expressing the glycoprotein gB (gB) of infectious laryngotracheitis virus (ITLV) based on the full-length cDNA clone of the thermostable TS09-C strain. This thermostable rNDV, named rTS-gB, displayed similar thermostability, growth kinetics, and pathogenicity compared with the parental TS09-C virus. The immunization data showed that rTS-gB induced effective ILTV- and NDV-specific antibody responses and conferred immunization protection against ILTV challenge in chickens. The efficacy of rTS-gB in alleviating clinical signs was similar to that of the commercial attenuated ILTV K317 strain. Furthermore, rTS-gB could significantly reduce viral shedding in cloacal and tracheal samples. Our study suggested that the rNDV strain rTS-gB is a thermostable, safe, and highly efficient vaccine candidate against ILT and ND.

## 1. Introduction

Infectious laryngotracheitis virus (ILTV) is an alpha herpesvirus causing upper respiratory tract disease in chickens and significant losses to the global poultry industry [1,2]. The severity of the disease varies from mild to acute, and the lethality rate can be as high as 70% [3]. Vaccination is a global strategy to control infectious laryngotracheitis (ILT) [4]. Although chicken embryo origin (CEO) or tissue culture origin (TCO) vaccines can significantly inhibit the outbreak of ILT, the safety of live attenuated vaccines is regarded as a challenging problem worldwide. Live attenuated vaccines can regain virulence and become the source of outbreaks [5,6].

Based on the limitations of the current live attenuated vaccines, new vaccines need to be developed, such as the live vector vaccine. However, compared with the live attenuated vaccines, the turkey herpesvirus (HVT) and fowl pox virus (FPV) vectors encoding ILTV antigens induce only partial protection [7]. Therefore, there is a significant need to develop safe and protective next-generation vaccines.

Newcastle disease (ND) is an important avian disease that seriously affects poultry worldwide and is caused by the Newcastle disease virus (NDV) [8,9]. Virulent NDV strains cause severe respiratory and neurological diseases in poultry. Currently, ND is mainly prevented and controlled through vaccination [10]. Naturally occurring avirulent NDV strains, such as the LaSota strain, can replicate efficiently in the allantoic cavity of chicken embryos, induce robust mucosal immunity in the respiratory system, and have been used as live vaccines worldwide [11]. At present, the NDV reverse genetic operating system is well-rounded. There are an increasing number of new vaccines using NDV as a vector to express the antigenic proteins of various avian viruses, such as the glycoproteins gB and gD of ILTV, the spike protein of infectious bronchitis virus, and the hemagglutinin protein of influenza virus [12,13,14]. These vaccines are highly effective in protecting chickens by eliciting humoral and cellular immune responses.

Thermostable avirulent NDV strains with thermostable and avirulent properties have the advantage of being administered in drinking water, sprays, and food and have been used as vaccines for the control of ND [15]. The thermostable NDV V4 strain was isolated from healthy chickens in Australia [16]. In previous studies, a thermostable avirulent TS09-C strain was developed by serial passage of the V4 strain in BHK-21 cells, and this strain has been used for the development of a thermostable NDV vaccine for the expression of a heterologous gene [17,18]. In this study, a TS09-C strain expressing glycoprotein gB of ILTV was developed, and the thermostability, pathogenicity, and immunogenicity of the TS09-C-vectored ILTV vaccine strain were evaluated in vitro and in vivo.

## 2. Materials and Methods

### 2.1. Cells and Viruses

BHK-21 cells were obtained from the China Center for Type Culture Collection (Wuhan, China). Cells were maintained in Dulbecco’s modified Eagle medium (DMEM) and incubated at 37 °C in 5% CO_2_. The ILTV strain WG, the NDV strain TS09-C and LaSota were maintained in our laboratory. The GenBank accession numbers of the ILTV strain WG and the NDV strains TS09-C and LaSota are JX458823, JX110635, and JF950510, respectively. All viruses were propagated in specific-pathogen-free (SPF) embryonated chicken eggs.

### 2.2. Plasmid Construction and Viral Rescue

The full-length cDNA clone of the NDV TS09-C strain, named pTS09-C, was used as a backbone for the insertion of the ILTV-gB gene between the P and M genes to generate pTS-gB. Briefly, the gB open reading frame (ORF) of the ILTV strain WG was synthesized by Sangon Biotech. Then, pTS09-C was polymerase chain reaction (PCR) linearized at the 5′-NTR of the M gene by using Pfu Ultra Fusion HS DNA polymerase (Agilent, Santa Clara, CA, USA) and vector-specific primers to include the complete sequence of this clone. The gB fragment fused with the sequence of the gene end (GE), gene start (GS), and Kozak was amplified by overlapping PCR and ligated with linearized pTS09-C using an In-Fusion PCR Cloning Kit (TaKaRa, Shiga, Japan). The sequences of all primers used in this study are available upon request.

The recombinant NDV rTS-gB was rescued as previously described [18]. Briefly, MVA-T7-infected BHK-21 cells were cotransfected with the full-length cDNA clone pTS-gB and three support plasmids expressing the NP, P, and L proteins using Lipofectamine 2000 (Invitrogen, Waltham, MA, USA) according to the manufacturer’s protocol. At 6 h posttransfection, the cells were washed with phosphate-buffered saline (PBS) and cultured in DMEM with 2% fetal bovine serum (FBS), antibiotics, and 0.2 μg/mL TPCK-trypsin (Sigma-Aldrich, St. Louis, MO, USA) for 72 h. The cell lysates were collected by freeze/thawing three times and centrifuged, and the supernatants were then inoculated into 10-day-old SPF embryonated chicken eggs. After 96 h of inoculation, the allantoic fluids were collected, and the recombinant viruses were identified by hemagglutination assay (HA) and reverse transcription PCR (RT-PCR) assays.

### 2.3. Western Blotting and Immunofluorescence Staining

For western blotting, BHK-21 cells were infected with rTS-gB at a multiplicity of infection (MOI) of 0.1. The cell lysates were harvested at 48 h postinfection (hpi). The collected cell lysates were analyzed by western blotting using a specific anti-gB rabbit antibody and anti-NDV chicken antibody (prepared in our laboratory). For immunofluorescence staining, BHK-21 cells grown in six-well plates were infected with rTS-gB at an MOI of 0.1. At 48 hpi, the cells were fixed with 4% paraformaldehyde and washed three times with PBS. The fixed cells were blocked in PBS containing 1% BSA at 4 °C for 1 h and incubated with primary antibody (rabbit anti-gB and chicken anti-NDV antibodies) for 1 h at 37 °C and secondary antibody (Alexa Fluor 594-labeled goat anti-rabbit IgG and FITC-labeled goat anti-chicken IgG) (Thermo Fisher Scientific, Waltham, MA, USA) for 1 h at 37 °C with PBS containing 1% BSA. Cells were stained with DAPI and then imaged under a confocal microscope (Leica, Wetzlar, Germany) using LAX X software version 3.3.2 (Leica Microsystems, Wetzlar, Germany).

### 2.4. Viral Titration and Growth Kinetics

The titers of rTS-gB were determined by the HA assay, the 50% tissue culture infectious dose (TCID_50_) assay in BHK-21 cells, and the 50% egg infectious dose (EID_50_) assay in 10-day-old SPF embryonated chicken eggs. To test the viral growth kinetics, monolayers of BHK-21 cells were infected with rNDVs at 0.1 MOI for 2 h, washed three times with PBS, then cultured with medium containing 2% FBS. The supernatants of cell culture were harvested at the indicated time points, and the viral titers in the supernatants were measured using the TCID_50_ assay.

### 2.5. Pathogenicity and Thermostability

The pathogenicity of the rNDVs was evaluated by measuring the mean death time of the minimal lethal dose (MDT/MLD) in 10-day-old SPF embryonated chicken eggs, and the intracerebral pathogenicity index (ICPI) in 1-day-old SPF chickens as previously described [17].

The viral thermostability was tested as described previously [18,19]. For thermostability at 56 °C, aliquots of 1.0 mL undiluted allantoic fluids infected with rNDVs were sealed in airtight vials and submerged into a water bath at 56 °C. At the indicated time points, the vials were transferred to an ice-cold water bath to stop the heat treatment. For the assessment of thermostability at 37 °C, aliquots of diluted allantoic fluids infected with rNDVs were incubated at 37 °C for 0, 1, 3, 7, and 14 days. The parental rTS09-C strain and the thermolabile LaSota strain were used as controls. The residual viral infectivity and HA activity associated with the heat-treated rNDVs were measured by HA assay and TCID_50_ assay, respectively. The decreased HA activity and infectivity of these viruses are shown on a logarithmic scale as a function of the heat treatment time. Regression lines were plotted from 4 or 5 time points.

### 2.6. Immunization and Challenge Experiments

Embryonated chicken eggs from SPF chickens were purchased from Beijing Boehringer Ingelheim Biotechnology Company (Beijing, China), hatched, and raised at the animal center of our institute. Forty-five SPF chickens (4 weeks old) were randomly allocated into three groups (15 birds per group), and one group was inoculated with rTS-gB (10^7.0^ TCID_50_/mL, 100 μL per bird) via the intranasal and intraocular (IN/IO) routes. The birds in the other two groups were inoculated with 100 μL of ILTV attenuated vaccine (K317 strain) and 100 μL of PBS as positive and negative controls, respectively, via the IN/IO route. Blood was collected from each bird at 7 and 14 days after immunization. Each group was then challenged with 1.0 × 10^4.0^ EID_50_ of the ILTV WG strain via the IN/IO routes. These birds were monitored daily for clinical signs and mortality for 15 days. Body weights were measured at various time points prior to challenge and 10 days postchallenge (dpc).

### 2.7. Serum Antibody Titers

Serum antibody titers were determined by enzyme-linked immunosorbent assay (ELISA) and the hemagglutination inhibition (HI) assay as described previously [20,21]. For ELISA, the purified gB protein (prepared in our laboratory) was used for coating in 96 well plate at 4 °C overnight. Serum was added after blocking and washing. The HRP-labeled anti-chicken secondary antibody was then added, and the plate was washed three times. For visualization, TMB chromogenic solution was added to each well, and the absorbance at 630 nm was then measured using an ELISA reader 318C+ (Shanghai Peiou analytical instrument, Shanghai, China).

For the HI assay, 2-fold serial dilutions of immunized chicken sera (50 μL) were prepared, and each dilution was mixed with 4 HA units of NDV LaSota strain. After 30 min of incubation at room temperature (RT), 50 μL of 1% chicken red blood cells was added, and the mixture was incubated for 30 min at RT. The HI titer was calculated as the highest serum dilution that showed complete hemagglutination inhibition.

### 2.8. Shedding of ILTV in Tracheal and Cloacal Swabs

Tracheal and cloacal swabs were collected from experimental birds at 3, 5, and 7 dpc. The supernatant of the tracheal and cloacal swabs was inoculated into the chorionic villi allantoic membrane of 9-day-old SPF chicken embryos (0.2 mL per embryo), and five chicken embryos were used for each sample. The inoculated chicken embryos were incubated at 37 °C for 96 h. ILTV shedding was evaluated by observing the pox spots on the chorionic villi allantoic membrane and performing the PCR assay.

### 2.9. Quantification of ILTV Load in Tracheal Swabs

Tracheal swabs were collected at 3, 5, and 7 dpc for viral detection using a quantitative real-time PCR (qPCR) assay as described previously [22,23]. A standard curve was created, starting with 3.56 × 10^10^ copies/µL for the gC plasmid. Serial 10-fold dilutions of the plasmid (from log_10_ 1.0 to 8.0) were prepared. qPCR assays were performed on a Light Cycler 96 (Roche, Basel, Switzerland). The reactions were prepared in a final volume of 20 μL as follows: 10 μL of 2 × SYBR qPCR master mix, primers at a final concentration of 0.25 µM, and 2 μL of DNA template. The reaction cycling profile used was 95 °C for 5 min, 40 cycles of 95 °C for 10 s, and 60 °C for 30 s. The cycle threshold (CT) value of the sample was converted into a copy number by substituting it into the standard curve. Light Cycler software was used to generate the standard curve and calculate the correlation coefficient of the standard curve.

## 3. Results

### 3.1. Generation of Recombinant NDV rTS-gB

**Figure 1:**
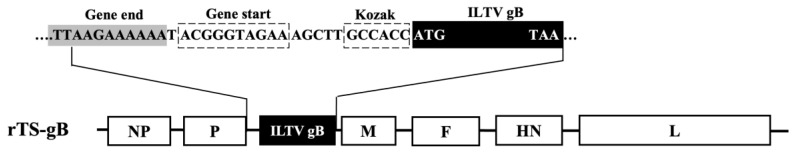
Schematic representation of the genome of the rNDV strain rTS-gB. The gB gene of ILTV was inserted into the genome of NDV TS09-C between the P and M genes. The NDV gene end and gene start signal sequences, Kozak sequences, and ILTV-gB gene were boxed.

To generate recombinant NDV expressing the gB protein of ILTV, the gB gene of the ILTV strain WG was synthesized and then inserted into the infectious cDNA clone of the thermostable avirulent NDV strain TS09-C (Figure 1).

**Figure 2:**
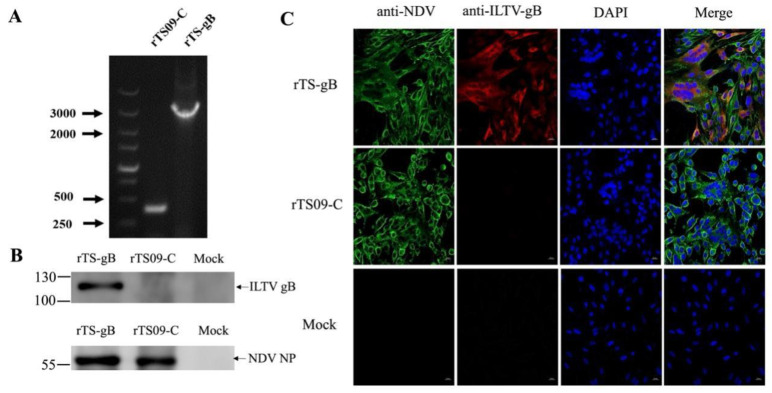
Identification of recombinant NDV expressing the gB protein of ILTV. (**A**) The insertion of the gB gene into rTS09-C was confirmed by RT-PCR using a pair of primers spanning the insertion region of the ILTV-gB gene. (**B**) BHK-21 cells were infected with rTS-gB or rTS09-C at an MOI of 0.1. The cells were harvested and lysed at 48 hpi, and the cell lysates were analyzed by western blotting using anti-gB or anti-NDV antibodies. (**C**) BHK-21 cells were infected with rTS-gB or rTS09-C at an MOI of 0.1. At 48 hpi, the cells were subjected to indirect immunofluorescence staining using anti-gB or anti-NDV antibodies. The cells were analyzed by confocal microscopy using ZEN 2.3 Lite software (Zeiss, Germany).

The recombinant virus rTS-gB was rescued from the transfected BHK-21 cells and propagated in SPF-embryonated chicken eggs. The presence of the inserted gB gene in rTS-gB was confirmed by RT-PCR (Figure 2A) and sequencing. The expression of the ILTV-gB or NDV NP protein in BHK-21 cells infected with rTS-gB and rTS09-C at 48 hpi was identified by western blotting. The expression of both the gB and NP proteins was detected in rTS-gB-infected cells, while only the NDV proteins were detected in the rTS09-C-infected cells (Figure 2B). Similar results were obtained by immunofluorescence staining. (Figure 2C). Our results confirmed that the rNDV strain rTS-gB was rescued successfully and could efficiently express the ILTV-gB protein in BHK-21 cells.

### 3.2. Biological Characteristics of rTS-gB

**Figure 3:**
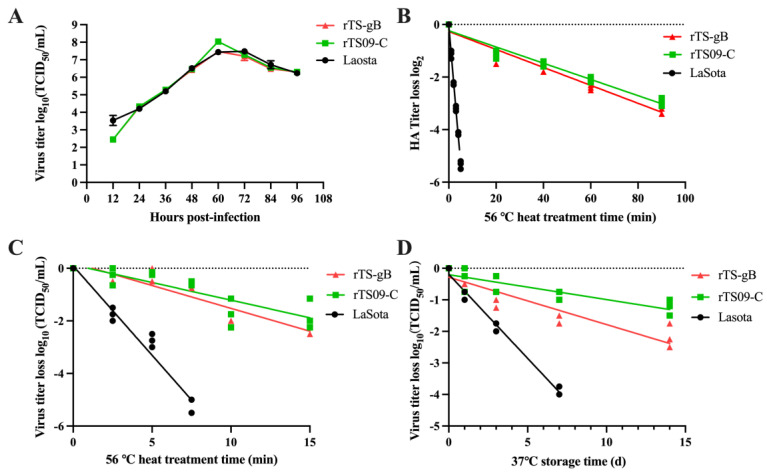
The growth kinetics and thermal properties of rTS-gB. (**A**) BHK-21 cells infected with rNDVs at MOI of 0.1 were harvested at the interval of 12 h, and the viral titers of cultured supernatants were determined using the TCID_50_ assay. After heat treatment at 56 °C, the HA titer (**B**) and viral titer (**C**) of heat-treated virus were determined by the HA assay and TCID_50_ assay. (**D**) At 37 °C preservation, the recombinant NDVs harvested at different time points were titrated using the TCID_50_ assay.

To determine whether the insertion of the gB gene into the NDV TS09-C genome affected the viral biological properties, the pathogenicity and growth kinetics of the rTS-gB virus were examined in vitro and in vivo by conducting titration assays, MDT/MLD and ICPI tests. As shown in Table 1, rTS-gB retained the low pathogenicity of the parental rTS09-C virus, with MDT values >168 h and ICPI values of 0.0. The viral titer of rTS-gB was slightly lower than that of rTS09-C in SPF chicken embryo eggs. Similarly, rTS-gB, rTS09-C, and LaSota reached peak titers of 10^7.5^, 10^8.0^, and 10^7.4^ TCID_50_/mL at 60 hpi, respectively (Figure 3A). rTS-gB showed a slightly lower viral titer than rTS09-C in BHK-21 cells. After heat treatment at 56 °C, the mean times for a 1 log_2_ decrease in the HA titers of rTS09-C and rTS-gB were 25 and 20 min, respectively; in contrast, the mean times for a 1 log_2_ decrease in the HA titer of the LaSota strain was 2.5 min (Figure 3B). For viral titer loss, the mean times for a 1 log_10_ decrease of rTS09-C and rTS-gB were much longer than that of LaSota (Figure 3C). These results demonstrated that the rNDV strain rTS-gB had high propagation efficiency in chicken embryos and was avirulent and thermostable.

The stability of rTS-gB was further assessed stored at 37 °C. Allantoic fluids containing rTS-gB, rTS09-C, and LaSota were stored at 37 °C from 1 to 14 days, and the residual titer of the virus at the indicated time points was determined in BHK-21 cells. At 7 days post-storage, the viral titer losses of the rTS-gB and rTS09-C strains were 10^1.5^ and 10^0.5^ TCID_50_/mL, respectively, whereas the viral titer loss of the LaSota strain was 10^4.0^ TCID_50_/mL (Figure 3D). The results showed that insertion of the gB gene did not greatly affect the thermostability of rTS09-C.

### 3.3. Immunogenicity of rTS-gB

**Figure 4:**
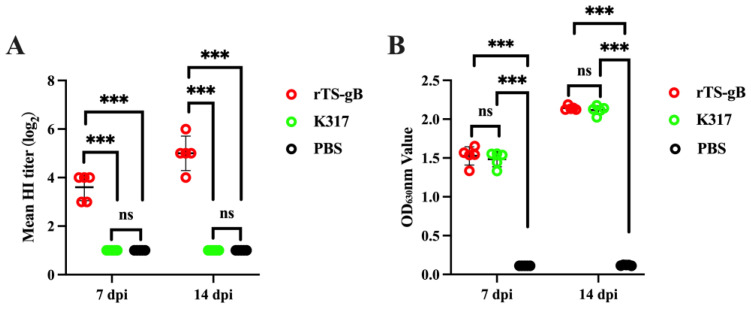
Humoral immune response induced by rTS-gB in chickens. Serum was collected from each bird at 7 and 14 dpi and used for antibody detection. (**A**) The NDV antibody was detected by HI assay. (**B**) The ILTV antibody was detected by ELISA. The data shown are the measured values of five chickens in each group. *** *p* < 0.001. ns, not significant.

To assess the humoral immune response induced by rTS-gB in chickens, we administered a single-time vaccination using rTS-gB (10^6.0^ TCID_50_/bird) via the IN/IO route and detected the NDV and ILTV antibodies of the experimental birds at 7 and 14 dpi. The serum antibody titers against ILTV and NDV were determined by ELISA and HI assays, respectively. The data showed that immunization with rTS-gB induced high levels of NDV- and ILTV-specific antibody responses in chickens (Figure 4A,B). Comparable ILTV antibody levels were observed between the rTS-gB and K317 vaccine groups (*p* > 0.05) (Figure 4B). Our results suggested that rTS-gB has similar immunogenicity to K317.

### 3.4. Protective Efficacy of rTS-gB against ILTV Challenge in Chickens

**Figure 5:**
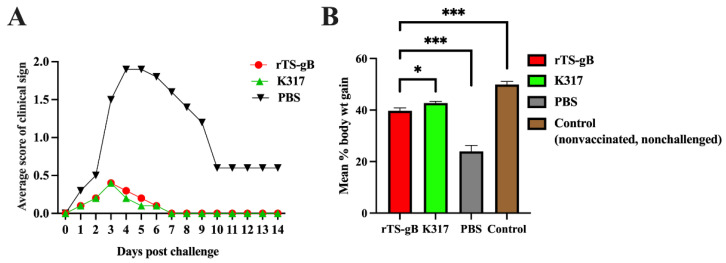
Clinical protection of rTS-gB against virulent ILTV challenge in chickens. Four-week-old SPF chickens were vaccinated with rTS-gB, K317, or PBS and challenged with the virulent ILTV WG strain at 14 dpi. The clinical signs were recorded daily, and the body weights were measured prior to challenge and at 10 dpc. The average clinical scores for each group of chickens (**A**) and the percentage of weight gain from prior to challenge to 10 dpc (**B**) were plotted. * *p* < 0.05; ** *p* < 0.01. *** *p* < 0.001. ns, not significant.

After challenged with the virulent ILTV strain WG, SPF chickens in the PBS group showed typical clinical signs from 2 dpc, such as depression, conjunctivitis, or death, scoring from 1 to 3 depending on the severity of disease symptoms (Figure 5A). The birds in the PBS group showed a peak in clinical signs between 3 and 6 days with a mortality rate of 10% to 20%, after which the severity of the symptoms gradually decreased. In contrast, more than 90% of the rTS-gB or K317 vaccinated chickens showed no disease signs, and only 2 to 5 birds displayed very mild clinical signs at 2 to 7 dpc in these groups. Moreover, ILTV infection can also lead to poor weight gain [24]. The percentage of weight gain in the rTS-gB group was similar to that of the K317 group and slightly lower than that of the control (nonvaccinated, nonchallenged) group but significantly higher than that of the PBS group (Figure 5B). Our results demonstrated that rTS-gB could significantly reduce clinical signs and weight loss and provide good protection against virulent ITLV challenge in chickens, which was comparable to that obtained with the K317 vaccine strain.

### 3.5. Reduction in the Shedding of Virulent ILTV in Challenged Chickens

The reduction in virus shedding after the challenge is another important criterion of vaccine efficacy. The shedding of ILTV from tracheal and cloacal swabs of challenged birds was detected by virus isolation in chicken embryos. As shown in Table 2, rTS-gB significantly reduced viral shedding in cloacal swabs from 3 dpc and tracheal swabs from 5 dpc. For tracheal swabs, the rate of virus shedding in both the TS-gB and K317 groups was 20% (1/5), whereas that in the PBS group was 66.7% (2/3) at 7 dpc. For cloacal swabs, the challenged virus could not be detected from 5 pdc in either the TS-gB or K317 groups, whereas the rate of virus shedding in the PBS group was 66.7% at 7 dpc.

**Figure 6:**
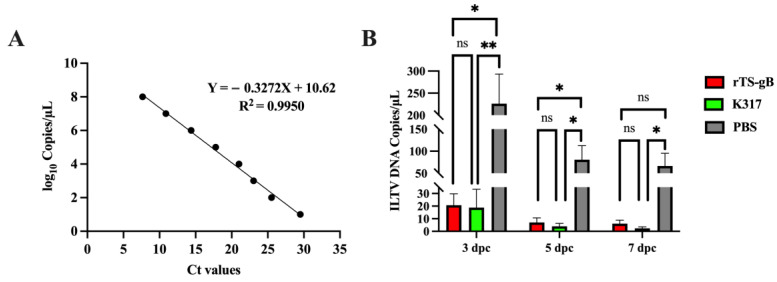
Load of shedding virus in vaccinated birds after virulent ILTV challenge. (**A**) Standard curve for the amplification of ILTV gC plasmid with increasing concentrations. (**B**) The load of shedding virus in tracheal swab samples. The data shown are the values from five swab samples in each group. * *p* < 0.05; ** *p* < 0.01. ns, not significant.

To further compare the load of shedding virus among these groups, the genome copies of virulent ILTV in tracheal swabs were measured by qPCR. The standard curve of ILTV qPCR was Y = −0.3272X + 10.62 (Figure 6A). At 3 dpc, the concentration of viral genome copies in tracheal swabs of the rTS-gB group was 11-fold lower than that of the PBS groups and comparable with that of the K317 group. Similar results were observed at 5 and 7 dpc (Figure 6B). These data confirmed that immunization with rTS-gB could greatly reduce the shedding of challenged virus in chickens.

## 4. Discussion

ILT and ND are two important avian diseases that cause significant economic losses to the poultry industry worldwide. The coinfection of ILTV and NDV could cause severe and irreversible damage to chickens [25]. Currently, the ILTV attenuated vaccine and lentogenic NDV vaccine are utilized to control ILT and ND, respectively [26]. However, the ITLV attenuated vaccines can regain virulence and become the source of outbreaks [24,27]. Recombinant viral vectors have been considered safe alternatives. The ILTV vaccines based on the FPV and HTV vectors can reduce the clinical signs but not the viral shedding after challenge, particularly in tracheal swabs [28,29]. The ILTV vaccine based on the thermolabile NDV LaSota vaccine strain has been proven to provide excellent protection and greatly diminish the viral shedding against ILTV [12].

By utilizing reverse genetic technology, many NDV-vectored vaccines have been developed. For example, a recombinant NDV expressing the spike protein of SARS-CoV-2 elicited potently neutralizing antibodies against SARS-CoV-2 in mice [30]. The rNDV coexpressing HIV-1 Env and Gag proteins elicited potently humoral, mucosal, and cellular immune responses in guinea pigs [31]. However, most of these studies used the thermolabile NDV strains as vectors. Previously, we developed a novel NDV vector based on the thermostable TS09-C strain, which requires at least 90 min for a 3 log_2_ (87.5%) decrease in HA activity and 15 min for a 1 log_10_ (90%) decrease in infectivity when heat-treated at 56 °C [18,32]. Here, we described the development of a live, thermostable NDV-vectored ILTV-gB vaccine strain and its evaluation for the pathogenicity, immunogenicity, and protective efficacy in SPF chickens.

Most vaccines are heat sensitive and therefore strictly rely on the cold chain to maintain their quality. Thermostable vaccines that are less dependent on the cold chain can greatly expand the global coverage of vaccines by reducing costs, ensuring efficacy, and reducing waste [33]. Here, the results of the thermostability test showed that rTS-gB could remain infectious for more than 14 days at 37 °C. These results indicated that rTS-gB could relieve cold chain transport bottlenecks and make it more suitable for drinking water and spray immunization.

gB and gD are the two major envelope glycoproteins that mediate the attachment of ILTV to the host cell receptor. The gD of alpha-herpesviruses involved in viral attachment to the host cell surface, and gB helped in the fusion of the viral envelope to the host cell membrane [34,35]. The HVT vector vaccine expressing gD and gI was more effective than the FPV vector vaccine expressing gB and membrane-associated protein reducing clinical signs and viral loads in the trachea [7]. However, the ILTV loads in the trachea of chickens vaccinated with the HVT or FPV vector vaccine were significantly higher than that of chickens vaccinated with the CEO vaccine after the challenge [7,36]. In the studies of NDV vector vaccines, the LaSota was used as a backbone in which the inserted gB was fused to the last 12 amino acids of the NDV F protein cytoplasmic tail, and the inserted ectodomain of gD was fused to the transmembrane and cytoplasmic tail of the NDV F protein. The LaSota expressing gD showed higher protective potency than the virus expressing gB due to the higher levels of envelope binding and surface expression of gD in infected cells [37]. Interestingly, the complete ORF of gB or gD was inserted into LaSota, and the LaSota expressing gB was better than the virus expressing gD in reducing the amount of virus shed in the eye and tracheal samples [12]. The presence of maternal antibodies to NDV and ILTV did not significantly interfere with the ability of the LaSota strain-vectored gB to elicit protective immunity against ILT [38]. Here, immunization with rTS-gB reduced both the severity of the disease and the replication of the challenge virus in the trachea. Compared with the unvaccinated birds, the chickens immunized with rTS-gB and K317 showed 10.9- and 12.1-fold reductions in virus shedding, respectively. The rTS-gB showed high immunogenicity, which was similar to that found with the ILTV commercial attenuated vaccine strain K317.

The rNDV strain rTS-gB has many advantages as a live vaccine. (1) rTS-gB can propagate efficiently in chicken embryos, and the vaccine is easy to produce. (2) rTS-gB is safe and can avoid regaining virulence. (3) rTS-gB can be used for controlling both ND and ILT. Although no NDV challenge test was performed in this study, the efficacy of protection against virulent NDV after immunization with rTS09-C recombinant virus expressing other exogenous genes in our previous studies was 100% [39]. Moreover, the average NDV HI antibody titer at 14 dpi in the rTS-gB group was higher than 4.0 log_2_, which was considered a completely protective level [18,39].

In summary, a recombinant NDV rTS-gB expressing the gB protein of ILTV was constructed and rescued using the thermostable avirulent strain TS09-C as a vector. The recombinant NDV rTS-gB was a good thermostable bivalent vaccine candidate for the rapid, efficient, and economical immunization of chickens against both ND and ILT.

## Figures and Tables

**Table 1:** Biological characteristics of the recombinant viruses.

Virus	Viral Titer (EID_50_/mL)	MDT/MLD * (h)	ICPI **
rTS-gB	10^8.80^	>168	0.0
rTS09-C	10^9.20^	>168	0.0

* MDT/MLD (the mean death time of minimal lethal dose) for 10-day-old SPF embryonated chicken eggs. ** ICPI (intracerebral pathogenicity index) for 1-day-old SPF chickens.

**Table 2:** Viral shedding in vaccinated chickens after virulent ILTV challenge.

Days Postchallenge	rTS-gB		K317		PBS	
	Tracheal Swabs	Cloacal Swabs	Tracheal Swabs	Cloacal Swabs	Tracheal Swabs	Cloacal Swabs
3	4/5	2/5	4/5	2/5	5/5	5/5
5	2/5	0/5	3/5	0/5	3/4	3/4
7	1/5	0/5	1/5	0/5	2/3	2/3

## Data Availability

Not applicable.
